# Chest pain and paralysis after pulse prednisolone therapy an unusual case presentation of thyrotoxic periodic paralysis: a case report

**DOI:** 10.4076/1757-1626-2-7501

**Published:** 2009-08-25

**Authors:** Stefan Hagel, Tereza Elznerova, Wenke Dietrich, Thomas Schrauzer, Stefan John

**Affiliations:** 1 Department of Nephrology and Hypertension, University Hospital Erlangen-NurembergBreslauerstrasse 201, 90471 NurembergGermany; 2 Department of Neurology, Nuremberg Municipal Academic HospitalBreslauerstrasse 201, 90471 NurembergGermany

## Abstract

Thyrotoxic periodic paralysis is a disease characterized by recurrent episodes of paralysis and hypokalemia during a thyrotoxic state. Thyrotoxic periodic paralysis is a common complication of hyperthyroidism in Asian populations, but can affect other ethnic groups as well. Due to population mobility, Thyrotoxic periodic paralysis is increasingly common in Western countries. Early diagnosis and prompt treatment of the thyrotoxic state and potassium supplementation prevent life-threatening complications associated with hypokalemia and muscle weakness. We present a young Turkish man who developed acute flaccid paralysis after receiving pulse prednisolone therapy for treatment of Pityriasis versicolor. His muscle strength and serum potassium fully recovered after potassium replacement and treatment of the thyrotoxic state which was a consequence of underlying Graves’ disease.

## Introduction

Acute tetraparesis is a neurological emergency which requires immediate diagnostic workup. Differential diagnosis of acute tetraparesis includes paraplegia caused by trauma, Guillain-Barré syndrome, ischemia, myasthenia gravis, inflammation, tumor, dissociative paralysis and periodic paralysis [1]. The heterogeneous group of muscle diseases known as periodic paralyses (PP) is characterized by episodic, sudden onset, flaccid paralysis of a single, several or all skeletal muscles with usually complete recovery between the attacks. Commonly, PPs are divided into primary, due to a genetic defect with familial or sporadic occurrence, and secondary forms due to drugs, suprarenal gland disease and misuse of laxatives or thiazide diuretics [2]. One primary form is the thyrotoxic periodic paralysis (TPP) which is characterized by recurrent episodes of paralysis and hypokalemia in the setting of thyrotoxicosis.

## Case presentation

A 32-year-old Turkish man was admitted to the emergency department because of severe intrascapular back pain, an episode of unconsciousness and weakness of both legs. Bilateral proximal muscle weakness of the lower extremities had started approximately 4 hours before admission and progressed rapidly. Three hours after appearance of the weakness the patient collapsed and was unconscious for 2 minutes, accompanied by urinary incontinence. No seizure was observed. After regaining consciousness he complained about severe intrascapular back pain, stabbing in nature without radiation. Initially the blood pressure was 80/60 mmHg, the heart rate was 100 bpm and the blood glucose level was 223 mg/dl. Emergency treatment with rapid infusion of cristalloid fluids was started and the patient was transferred to the hospital. When the patient arrived at the emergency department the blood pressure was 110/60 mmHg and the heart rate was 120 bpm. On his trunk and arms were confluating erythematous patches which appeared 3 days ago. Body temperature was normal. His muscle strength was found to be 2/5 on the MRC scale (Medical Research Council Paralysis Scale) in both lower extremities and 3/5 in both upper extremities. The deep tendon reflexes were somewhat diminished, otherwise neurological and physical examination were normal. He smoked 50 cigarettes a day and his alcohol intake was moderate. The family history was negative for cardiovascular disease. Beside nephrectomy after traumatic kidney rupture in childhood no relevant previous disease was present and no previous muscle weakness was noticed. However he reported that he consulted his GP the same morning for treatment of the erythematous patches. The GP gave him a pulse i.v. corticosteroid infusion (250 mg prednisolone).

**Figure 1.**
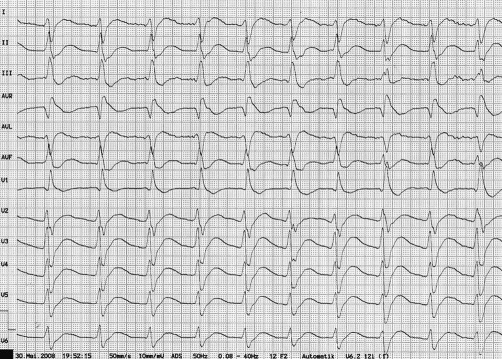
Electrocardiogram showing sinus tachycardia, a prolonged QTc interval, ST-segment depressions in all leads and T-U-wave complexes in the lateral leads.

The electrocardiogram revealed a sinus tachycardia of 118 bpm, a prolonged QTc interval, ST-segment depressions in all leads and T-U-wave complexes in the lateral leads (Figure 1). To rule out an aortic dissection a contrast computed tomography of the chest was performed immediately after admission, however no structural abnormality was detected. The only noticeable finding was a severely reduced cardiac output, recognized at the delayed contrast of the arteries. The biochemical laboratory analyses were within normal limits, except of a serum potassium level of 1.2 mmol/l [Norm 3.5-5.0 mmol/l], a ph of 7.26 [Norm 7.37-7.45], a bicarbonate level of 16.9 mmol/l [Norm 22-30 mmol/l], a base excess of -9.0 mmol/l [Norm -2.0-3.0 mmol/l] and a chloride level of 116 mmol/l [Norm 95-105 mmol/l], a TSH (Thyroidea stimulating hormone) level of <0.01 µIU/ml [Norm 0.27-4.20 µIU/ml], a fT3 (free trijodthyronine) level of 10.82 pg/ml [Norm 2.53-4.34 pg/ml] and a fT4 (free thyroxine) level of 3.2 pg/ml [Norm 0.9-1.7 pg/ml]. After receiving the laboratory results TPP was diagnosed and intravenous administration of potassium chloride was begun. Because of prior contrast medium exposure a therapy with methimazol and perchlorate was started. After administration of 30 mmol of potassium chloride within 3 hours, the patient got serious cardiac bradyarrhythmias followed by an asystolia which required mechanical reanimation for a short period of time. The serum potassium level at that time was 1.6 mmol/l. During the following 4 hours the patient developed a rebound hyperkalemia with a potassium level of 9 mmol/l which however did not induce any complications and lasted for 60 minutes despite any medical interventions. After intravenous administration of overall 90 mmol of potassium chloride the patients muscle strength and serum potassium were fully restored within 12 hours. Further laboratory diagnostics showed a high titre of thyrotropin-receptor antibodies, [7,4 IU/l (Norm <1,0 IU/l)] and a high titre of thyroid peroxidase antibodies [540 IU/ml (Norm <34 IU/ml)], both providing evidence of Graves’ disease [3]. Ultrasound examination revealed a normal sized thyroid gland with an inhomogeneous pattern. The skin changes were diagnosed as Pityriasis versicolor.

## Discussion

This case report highlights an unusual presentation of a patient with symptoms consistent with those seen in patients with an aortic dissection, where further diagnostics however revealed an initial manifestation of TPP which was induced by intravenous pulse prednisolone therapy. TPP is a well-known complication of thyrotoxicosis in Asian populations. The overall incidence of TPP among patients with hyperthyroidism in China and Japan is 1.8 and 1.9% respectively [4-5], whereas it is 0.1-0.2% among North American population with thyrotoxicosis [6]. Recently *Cesur et al.* analyzed 40 cases of TPP within the Turkish population, where most of the population are Caucasoid, with the main subgroup belonging to the Mediterranid extraction [7]. This report and other reports from the Mediterranean regions indicate the tendency to find TPP in the Mediterranid ethnicity other than the remaining Caucasoid subspecies. Despite the woman predominance of hyperthyroidism, TPP occurs more commonly in men at a ratio of 20 ;: ;1. The majority of thyrotoxic patients associated with TPP is due to Graves’ disease, while other conditions have been reported [8]. TPP is characterized by recurrent, transient episodes of muscle weakness to complete flaccid paralysis in the setting of a low serum potassium level and biochemical evidence of thyrotoxicosis, low TSH along with elevated fT4 or fT3, as observed in our patient. Deep tendon reflexes are reduced or absent. Findings of thyrotoxicosis may be subtle or even clinical silent [9]. The pathogenesis of TPP remains still unclear. Hypokalemia is the consequence of a rapid and massive shift of potassium from the extracellular into the intracellular compartment, related to increased sodium-potassium-adenosine triphosphate (Na/K-ATPase) pump-activity [8]. Patients with TPP have significantly higher Na/K-ATPase pump number and activity than thyrotoxic patients without TPP and the activity returns to normal when their thyroid function is controlled. Correction of the underlying thyrotoxic state aborts the attacks, but they can recur with the return of thyrotoxicosis [4]. It has been shown that thyroid hormones increase via transcriptional and posttranscriptional mechanisms Na/K-ATPase activity in skeletal muscles. Apart from direct stimulation by thyroid hormones, catecholamine and the enhanced β-adrenergic response in thyrotoxicosis further increases the activity of the Na/K-ATPase in skeletal muscle [8]. Furthermore there are indications that insulin plays an important role in the development of TPP and it could be demonstrated that serum insulin levels are elevated prior to the attack. Insulin-response sequences are present in the upstream region of Na/K-ATPase genes in skeletal muscle and insulin has been shown to stimulate Na/K-ATPase activity which may explain the association of TPP attacks with carbohydrate-rich meals. Other precipitating factors for an attack are ingestion of alcohol or strenuous exercise [2]. Overall it appears that patients with TPP have an underlying predisposition for activation of Na/K-ATPase activity, either directly by thyroid hormone or indirectly via adrenergic stimulation, insulin or exercise. To determine whether this predisposition is genetically associated rigorous attempts have been devoted to the search of the gene mutation of ion channels in TPP. The pathogenesis of FHPP (Familial Hypokalemic Periodic Paralysis), another entity of the primary periodic paralyses which is frequently experienced in Caucasian countries [10], has been elucidated and in most cases the abnormal gene is the alpha-1 subunit of the dihydropyridine-sensitive calcium channel in skeletal muscle (CACN1AS). In others it appears to be due to mutations in the skeletal muscle sodium channel (SCN4A) or the potassium channel (KCNE3) [11]. In TPP however, to date only certain single-nucleotide polymorphisms (SNiPs) of CACN1AS were associated with TPP in southern Chinese. Those SNiPs may provide a risk to the attack of TPP [12]. Glucocorticoids may induce hypokalemia from a transcellular potassium shift caused by an increased Na/K-ATPase pool in skeletal muscles and steroid-induced hyperinsulinemia and hyperglycemia which we also observed in our patient [13-15]. Current treatment recommendations involve treating the underlying hyperthyroid state and supplementation with potassium chloride (KCL) to prevent major cardiopulmonary complications. The dose of KCL required varies between 40 and 200 mmol. However excessive potassium replacement may result in rebound hyperkalemia during recovery of the paralysis when potassium is released from cells as the paralysis subsides, posing another risk of cardiac arrhythmia [8]. Furthermore a therapy with nonselective β-blockers has been reported, based on the implication of hyperadrenergic activity in the pathogenesis of TPP [8]. Whether the combination of low dose KCL and nonselective β-blockers is the treatment of choice in facilitating the recovery and reducing rebound hyperkalemia awaits further study in future.

In conclusion, TPP may be seen not only in the Asian population, but also in the Turkish and also in the Caucasian populations, especially in the Mediterranid. It is important to be aware of features of TPP, although it is crucial to emphasize that other diagnostics like computed tomography, magnetic resonance imaging or cerebrospinal fluid puncture must not be delayed to exclude other serious diseases. Current treatment recommendations involve treating the underlying hyperthyroid state, KCL supplementation which should be given as small as possible (<10 mmol/hr) to avoid rebound hyperkalemia unless there are cardiopulmonary complications and β-blockade.

## Abbreviations

Bpm: beats per minute
CACN1AS: alpha-1 subunit of the dihydropyridine-sensitive calcium channel in skeletal muscle
FHPP: familial hypokalemic periodic paralysis
fT3: free trijodthyronine
fT4: free thyroxine
GP: general practitioner
KCL: potassium chloride
KCNE3: potassium voltage-gated channel, Isk-related family, member 3
MRC scale: medical research council paralysis scale
Na/K-ATPase: sodium-potassium-adenosine triphosphate pump
PP: periodic paralyses
SCN4A: sodium channel, voltage-gated, type IV, alpha subunit
SNiPs: single-nucleotide polymorphisms
TPP: thyrotoxic periodic paralysis
TSH: thyroidea stimulating hormone
